# Lipidomic Analysis to Assess Oxidative Stress in Acute Coronary Syndrome and Acute Stroke Patients

**DOI:** 10.3390/metabo11070412

**Published:** 2021-06-23

**Authors:** Martin Malý, Martin Hajšl, Kamila Bechyňská, Ondřej Kučerka, Martin Šrámek, Jiří Suttnar, Alžběta Hlaváčková, Jana Hajšlová, Vít Kosek

**Affiliations:** 1 Department of Medicine, First Faculty of Medicine, Charles University in Prague and Military University Hospital, U Vojenské Nemocnice 1200, 169 00 Prague, Czech Republic; martin.maly@uvn.cz (M.M.); martin.hajsl@uvn.cz (M.H.); ondrej.kucerka@uvn.cz (O.K.); 2 Department of Food Chemistry and Analysis, University of Chemistry and Technology, Technická 3, 166 28 Prague, Czech Republic; Kamila.Bechynska@vscht.cz (K.B.); jana.hajslova@vscht.cz (J.H.); 3 Comprehensive Stroke Center, First Faculty of Medicine, Charles University in Prague and Military University Hospital, U Vojenské Nemocnice 1200, 169 00 Prague, Czech Republic; martin.sramek@uvn.cz; 4 Institute of Hematology and Blood Transfusion, Prague, U Nemocnice 2094, 128 20 Prague, Czech Republic; Jiri.Suttnar@uhkt.cz (J.S.); Alzbeta.Hlavackova@uhkt.cz (A.H.)

**Keywords:** acute coronary syndrome, stroke, plasma, lipidomics, high-resolution mass spectrometry

## Abstract

Alterations in lipid metabolism mediated by oxidative stress play a key role in the process of atherosclerosis and superimposed thrombosis; these can lead to acute coronary syndrome (ACS) and acute ischemic stroke (AIS). Multiple studies have shown that the formation of atheromatous lesions is initiated by oxidation of low-density lipoproteins incorporated into the intima of the vessel wall. Here, we studied lipids in plasma samples from three cohorts: 61 patients with ACS (group A), 49 patients with AIS (group D), and 82 controls (group K). Untargeted lipidomics based on high-performance liquid chromatography coupled to mass spectrometry (UHPLC-HRMS) was employed to obtain comprehensive information on whether relationships exist between these patient categories based on lipid patterns. In addition, malondialdehyde (MDA) as a standard marker of oxidative stress was monitored. The most characteristic lipids in group K were fatty acyls of hydroxyfatty acids (FAHFAs). As expected, MDA concentrations were the lowest in group K. Our findings can better explain ongoing pathologies, both acute and chronic, with the potential for future diagnosis and treatment.

## 1. Introduction

Lipids are involved in many metabolic processes of living organisms and represent a heterogenous biochemical group. The main characteristic is a lack of solubility. The six major categories include fatty acyls, glycerolipids, glycerophospholipids, sphingolipids, sterol lipids, and prenol lipids [1]. Transport in plasma is enabled by forming lipoproteins [2]. The role of lipids in atherogenesis has been proven in many clinical trials [3,4,5].

The first phase of atherogenesis is endothelial dysfunction and is caused by several risk factors leading to impairment of the production of endogenous vasodilators and activation of the expression of adhesive molecules. Low-density lipoproteins (LDL) can be modified by metabolites of oxidative stress and penetrate through this dysfunctional endothelium from plasma to the vessel wall; the LDL are then internalized by macrophages to form foam cells. The resulting subclinical inflammation leads to enhanced endothelial dysfunction and accumulation of immune active cells in the vessel wall. Lipids, together with immunologically active cells, are the cornerstones of the formation and growth of atherosclerotic lesions. The growth of these plaques, their rupture, and subsequent superimposed thrombosis lead to abrupt closure of the vessel, resulting in acute coronary syndrome (ACS), acute ischemic stroke (AIS), or acute limb ischemia. These are leading causes of morbidity and mortality in high-income countries [6].

As mentioned above, there is clear evidence for the central role of plasma lipids in the development of cardiovascular disease [7]; nevertheless, the routine measurement of pathology predictors for risk stratification, such as total plasma triacylglycerols or cholesterol (LDL, HDL), provide only crude information on the patient’s status. The mammalian plasma lipidome consists of thousands of species—there are approximately 40,000 structures described in the LIPIDMAPS project [8].

Several studies dedicated to revealing altered metabolic pathways during ACS or other cardiovascular diseases have been published and have employed various analytical methods for the measurement of plasma/serum lipids. Compared with tissue samples, the collection of plasma is non-invasive; moreover, a wide range of metabolites circulating in the blood and originating in different body tissues is present. Most studies, however, focused only on subsets of the metabolome, i.e., the methods used were target metabolite specific and thus did not provide comprehensive information on possible changes in the metabolome that might have occurred. The technique often used to analyze serum/plasma is ^1^H NMR because it requires minimal sample handling prior to measurement, is nondestructive, and is easy to perform; however, it is harder to interpret [9,10]. ^1^H NMR is complementary to mass spectrometry (MS), which is applicable in relevant studies. In some cases, the plasma/serum metabolome is analyzed by GC-MS, which requires time-consuming derivatization of more polar metabolites such as amino acids or saccharides to make them sufficiently volatile [11,12,13]. The most comprehensive approach to studying metabolome changes associated with cardiological disorders [14], including those dealing with ACS [15], is high-performance liquid chromatography coupled to mass spectrometry (HPLC-MS).

Here, we focused on lipidome characterization in the plasma of patients with ACS and AIS. Advanced analytical UHPLC-HRMS strategies followed by multi-dimensional statistics for data assessment were used. The objective was to search for characteristic markers associated with studied pathologies. Malondialdehyde (MDA) is a standard marker of oxidative stress and was measured to assess its correlation with alterations in lipid patterns.

## 2. Results

The data generated by the optimized UHPLC-HRMS method were processed by advanced multi-dimensional statistics. The major outcomes are summarized below.

### 2.1. Multivariate Analysis for Data Overview

**Figure 1:**
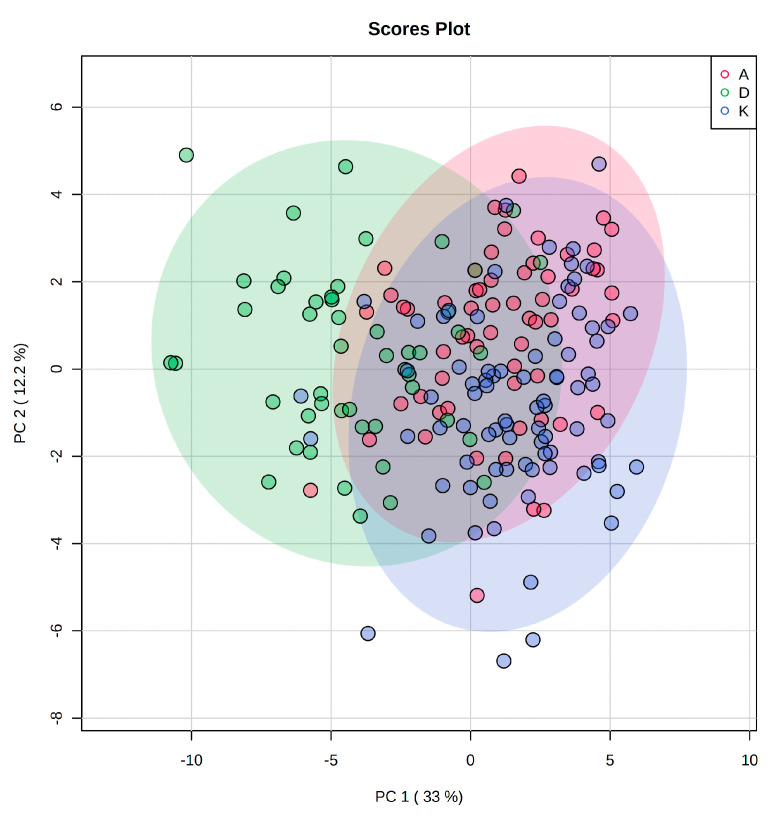
Clustering of sample groups in PCA based on data generated by LC-HRMS analysis of plasma sample sets (ANOVA-significant lipids only).

PCA score plots were the first step for variance analysis in QC samples. These were closely grouped, documenting good stability of the analytical system. In total, 438 lipid species in the samples were detected under the optimized instrumental setting. An overlap of two clusters of groups A and K was observed. In contrast, the group D cluster was partially separated from the other two (Figure 1). After the initial data evaluation, the compounds were filtered by ANOVA (*p*-value < 0.01, FDR corrected). Of the 438 features, 191 were statistically significant. This way, we could reduce the number of variables used for supervised statistical analysis and retain only the most important ones. 

### 2.2. Supervised Statistical Analysis of All Lipidome Components

Supervised statistical methods were applied on the matrix of statistically significant lipids only. A single PLS-DA model (R^2^X = 0.663, R^2^Y = 0.696, Q^2^Y = 0.531) for all three groups was built to search for the lipids that most strongly contributed to class separation. In total, 32 lipids achieved a VIP score higher than 1; their fold changes are shown in Table 1. The effect of lipoprotein lipase was identified as the main factor separating the groups after careful investigation of the list, as primarily TGs and their hydrolysis products FFAs (but not DGs and MGs) were found to be significant.

### 2.3. Analysis of Polar Lipids Subset

**Figure 2:**
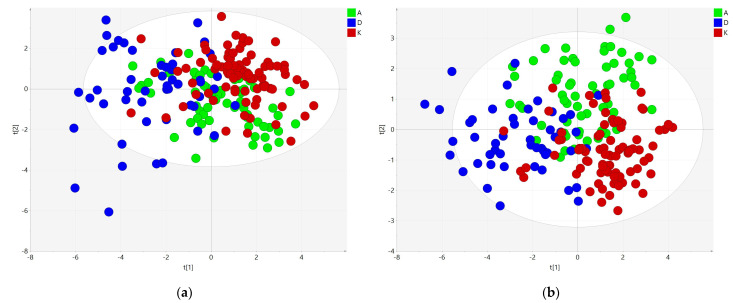
Explorative analysis on polar lipids subset. (**a**) PCA score plot showing clusters for all three groups (**b**) PLS-DA score plot.

To eliminate the effect of LPL enzyme, we removed neutral lipids (TGs, CEs) and their hydrolysis products (DGs and FFAs) from the data matrix and performed statistical analyses on the lipid species presumably not affected by LPL. They were defined as the polar lipid (PL) set. Similarly, as an entire lipidomic set, the PL set was filtered on ANOVA (*p*-value < 0.01) (Supplementary Table S1) and subsequently analyzed using PCA. Of 353 polar lipids, 84 were statistically significant and used for multivariate analysis (Figure 2). Similar groupings were observed, although tighter grouping of samples from the same group was observed. Thus, the PLS-DA model was built with medium efficiency (R^2^X = 0.699, R^2^Y = 0.649, Q^2^ = 0.521). However, this approach still facilitated searches for features that were significantly different in sample groups. 

In this analysis of the PL subset, we noticed changes in lipids not attributable to LPL; 28 lipids exceeded a VIP score > 1. The most pronounced differences seemed to be between groups D and K. In group D, lower levels of FAHFAs, plasmenyl PS, and several lysophospholipids were observed (usually containing polyunsaturated fatty acids with 20+ carbons).

**Figure 3:**
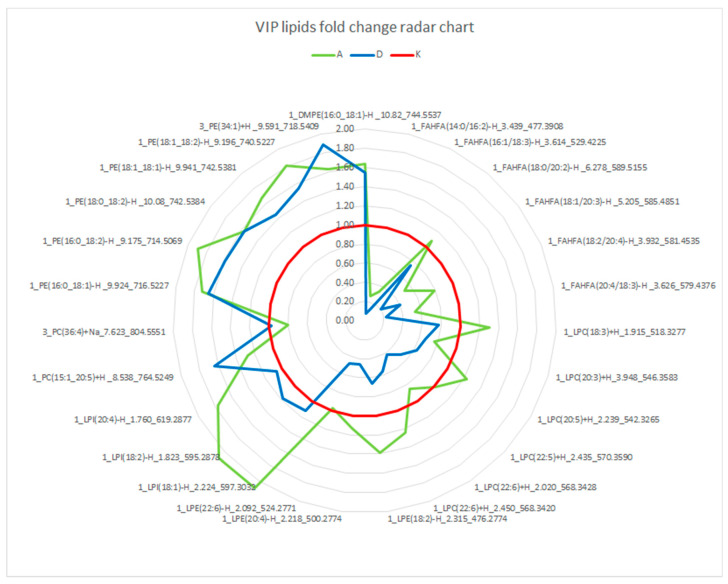
Radar chart showing trends of significant polar lipids among the groups by plotting the median fold change over the control group. FAHFA—fatty acyl of hydroxyfatty acid; LPC—lysophosphocholine; LPE—lysophosphoethanolamine; LPI—lysophosphoinositol; PC—phosphocholine; PE—phosphoethanolamine; DMPE—dimethylphosphoethanolamine.

The fold changes over group K were calculated with median values for each VIP compound (Table 2) and plotted in the radar chart (Figure 3). Most compounds differentiated group K and exhibited similar patterns in both groups A and D; there were some specific compounds for each group. Specifically, the number of LPI lipids increased in group A, and the number of LPCs and LPEs decreased in group D. The median fold changes over controls did not even increase to 2, except for LPI (18:2) and LPI (18:1); however, they decreased to values as low as 0.12 (8-fold decrease) in the case of FAHFA lipids. VIP polar lipids were also correlated with MDA concentrations; however, no correlation was seen by high Spearman coefficients despite most of the correlations showing low *p*-values. The highest absolute Spearman coefficients were −0.465 for FAHFA (18:2/20:4) and −0.433 for FAHFA (20:4/18:3).

### 2.4. The Effect of Prior Statin Use

**Figure 4:**
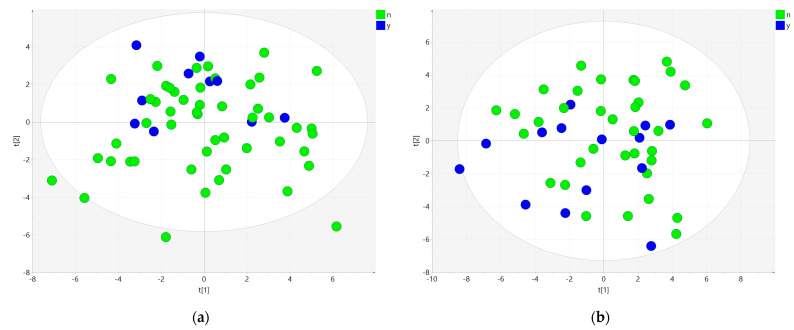
Explorative analysis on polar lipid subset. PCA score plots of samples from (**a**) group A and (**b**) group D. Samples are colored according to prior statin use (blue) or no prior statin use (green).

When inspecting the effects of prior statin use, we performed PCA on the profile of all polar lipids for both groups A and D (Figure 4). In both groups, the samples of patients with statin use were indiscernible from the samples of patients with no prior statin use.

### 2.5. Effect of Sex

It is a well-documented fact that ischemic heart disease manifests differently in males and females. There is a different clinical manifestation, exposure to risk factors, and a not completely understood protective role of hormones. In addition, males and females differ in the physiology of coronary circulation and cardiomyocyte function. The latter group has typically smaller coronary arteries, resulting in differences in shear stress and inflammatory mediators, resulting in more diffuse disease with less focal obstructive patterns. This diffuse character of the disease is associated with more pronounced vasomotor dysfunction rather than focal obstruction typical for males [16].

**Figure 5:**
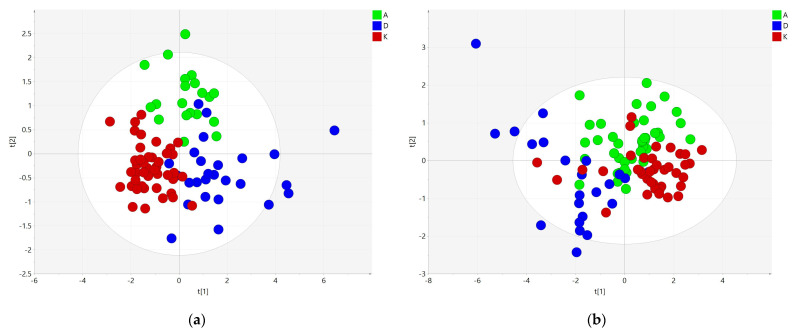
Supervised analysis on polar lipid subset. PLS-DA score plots of (**a**) female samples and (**b**) male samples. Group A in green, group D in blue, and group K in red.

Considering these facts, we split the dataset into male and female patient data and processed them separately in order to investigate possible differences in polar lipid patterns. The number of polar lipids filtered on ANOVA (*p*-value < 0.01) was 35 for females and 83 for males. While PCA score plots of the patients looked similar for both females and males (Figure 5), the markers differentiating the groups in each set were different, and quite surprisingly, of 37 unique VIP polar lipids for these models, only 7 were present in both of them: specifically, FAHFA (14:0/16:2), FAHFA (16:1/18:3), FAHFA (18:1/20:3), FAHFA (18:1/20:3), FAHFA (18:2/20:4), FAHFA (20:4/18:3), and FAHFA (14:0/16:2) along with LPC (20:1) and LPC (20:3). 

The markers separating groups in the female PLS-DA model (R^2^X = 0.501, R^2^Y = 0.575, Q^2^Y = 0.526) alongside FAHFAs were a number of different lipids. The highest importance was observed for LPIs such as in the whole sample set, plasmenyl PCs, and plasmenyl PSs that were not important for the whole lipid set. In addition, the number of LPCs, PCs, and PEs were important for the female model. For the male PLS-DA model (R^2^X = 0.729, R^2^Y = 0.426, Q^2^Y = 0.333), the important markers were a number of lysophospholipids, LPCs, and LPEs containing polyunsaturated fatty acids. All markers are summarized in Table 3 for the female model and Table 4 for the male model. 

### 2.6. Malondialdehyde Concentration Analysis

**Figure 6:**
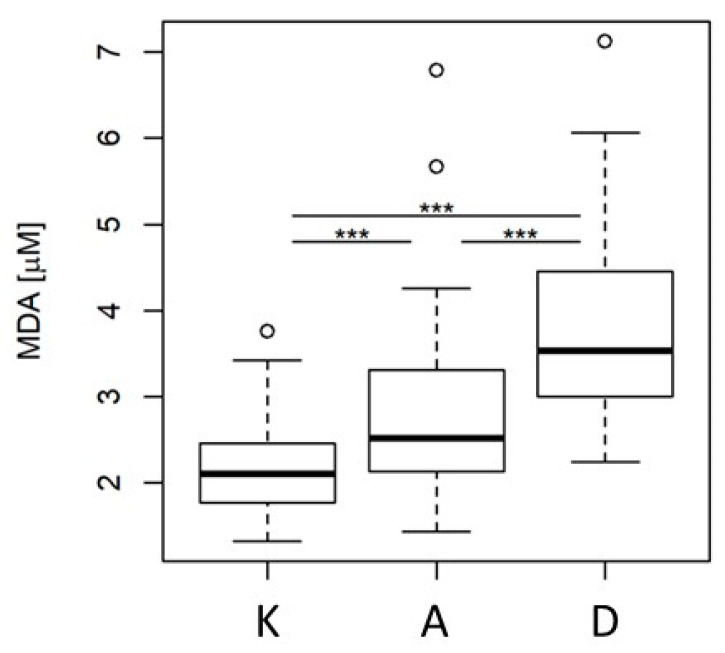
Box plot of MDA levels in patients’ plasma: A—acute coronary syndrome; D—acute ischemic stroke, K—control group. A vertical boxplot is constructed between the first (Q1) and the third (Q3) quartile, with the horizontal median inside. Data displayed as circles are outliers. *** *p* < 0.001 using post hoc Dunn’s test.

Plasma MDA was measured as a classic oxidative stress marker [17]. Based on the Kruskal–Wallis rank sum test, we found that concentrations of MDA were statistically different between the groups (Figure 6). The concentration of MDA increased in group A and further increased in group D. Post hoc Dunn’s test pointed to all three comparisons as significantly different. MDA levels were further used for correlation with VIP polar lipids.

## 3. Discussion

The LC-HRMS/MS technique detected and identified 438 lipid species; however, the statistical evaluation of the data must be carried out carefully. The statistical analysis of the whole lipidome revealed that the most distinctive lipids for the PLS-DA model are (i) multiple free fatty acids; (ii) fatty acyls of hydroxy fatty acids; (iii) lysophosphocholines (LPCs), lysophosphoethanolamines (LPEs), and lysophosphoinositols (LPIs); and (iv) triacylglycerols (TGs). The apparent importance of TGs and FFAs for the model can be explained by different type of heparins used (low-molecular-weight heparin (LMWH) in patient group D and unfractionated heparin (UFH) in patient groups A and K). As shown by Nasstrom, the hydrolytic activity of lipoprotein lipase (LPL) was released into the bloodstream after heparin administration and is higher in the case of UHF. When administering LMWH, the peak of LPL activity was only about 30%, and the subsequent plateau of LPL activity was only about 40% versus that seen with UFH [18]. LPL mainly hydrolyzes neutral lipids such as TGs and CEs, and these species, together with their hydrolysis products DGs and FFAs, were removed to form a new polar lipid dataset to avoid interpretation bias.

The statistical analysis of the polar lipid subset included 353 lipids, of which 84 were significantly different between the studied groups (FDR-corrected ANOVA *p*-value < 0.01). The supervised statistical analysis revealed 28 lipids to be significant in terms of a high PLS-DA VIP score. The lipids with the highest VIP scores were several representatives of FAHFAs and LPIs. FAHFAs were repeatedly attributed not only to insulin resistance but also to inflammation [19,20]. Higher levels in group K suggest increased anti-inflammatory activity, which is lower in group A and even lower in group D. Increased levels of LPI species in group A were observed and can be attributed to the release from platelets during thrombin activation [21]. Decreased levels of LPCs and LPEs were observed in the plasma of group D patients due to LPL and hepatic lipase (HL) exhibiting phospholipase activity [22]; HL has higher phospholipase activity than LPL [23]. However, the number of PEs and one PC (PC 15:1_20:5) showed higher plasma levels in both group A and D patients. This suggests that there is an increase in the phospholipids in group A, which has a bigger impact than hydrolysis. Given the fatty acid composition of LPEs, LPCs, PEs, and PCs, we speculate that polyunsaturated fats (PUFAs) containing phospholipids are the primary targets of LPL and HL activity. We assume that this is not the case for LPIs because they increase in group A and not in group K despite UHF administration to patients.

The effect of heparin treatment raises questions about whether other studies are affected. Interestingly, several studies report on differences in FFA or TG levels in ACS patients without mentioning the effects of hydrolytic enzymes in post-heparin plasma. For example, Lee et. al. showed that TGs are clearly decreased in lipoproteins of ACS patients, but the heparin effect is not mentioned [24]. Similarly, Ali et. al. demonstrated increased levels of several FFAs in the plasma of ACS patients without mentioning heparin [9]. We emphasize that great care must be taken when recruiting patients for the study because our prior treatment with heparin seems to be essential for the discovery of relevant biomarkers. Our control group was catheterized and received UHF as part of the procedure before catheterization. A similar approach was adapted by Meikle et al., where the HDL lipidome of catheterized CAD patients was compared to HDL lipidomes of ACS patients, and Yin et. al., where the plasma of ACS and non-ACS patients before PCI was analyzed (we assume both groups were administered heparin) [15,25]. These two studies show decreased levels of LPCs and LPEs in ACS patients’ plasma or high-density lipoproteins (HDL).

The effect of prior statin therapy was investigated as well. However, the data did not suggest any observable effect on the polar lipidome. The reason might be that plasma was collected before high-dose statin treatment at the hospital; therefore, no effect of this high dose of statins can be observed. The effect of sex was also investigated. When two separate sample sets were created and processed similarly, interesting patterns could be observed. While the distribution of lipids was similar in both females and males, the features separating the groups were different, suggesting different mechanisms at play in females and males. The only common features were FAHFAs and a pair of LPCs, which can be attributed to a common inflammation mechanism.

## 4. Materials and Methods

### 4.1. Study Design

We compared the plasma samples of three groups of patients: group A with ACS, group D with AIS, and the control group (group K) diagnosed in the Central Military Hospital University Military Hospital in Prague, Czech Republic (Table 5). In these samples, there were lipidomic data generated by optimized UHPLC-HRMS methods processed by advanced multi-dimensional statistics. The results were correlated with the MDA levels.

Regarding the HRMS lipidomic analysis, the molecular features were extracted by LipidMatch suite that relies on MZmine 2 software for extraction and the lipid identification based on an in silico fragmentation library search. The least class-specific fragment ions were required for lipid identification. Multivariate analysis by means of principal components analysis (PCA) was performed in both MetaboAnalyst and SIMCA for statistical evaluation of hundreds of features.

### 4.2. Chemicals and Materials

Methanol and 2-propanol were supplied by Merck (Darmstadt, Germany). Ammonium acetate, ammonium formate, formic acid, and acetic acid were obtained from Sigma-Aldrich (Prague, Czech Republic). Click Fit 2 mL Eppendorf tubes were purchased from TreffLab (Degersheim, Germany); 2 mL cryovials and autosampler vials were purchased from Labicom (Olomouc, Czech Republic).

### 4.3. Sample Collection

Blood samples were drawn from the patients of all three groups in vacutainer tubes containing EDTA and centrifuged immediately at 4000× *g* for 5 min at 4 °C. The plasma samples were stored in the dark at −80 °C until analysis.

We analyzed 61 plasma samples from patients undergoing PCI due to ACS. Blood samples were collected from an arterial sheath inserted in the radial or femoral artery, depending on intervention. UFH was administered 60 min before blood sampling; 49 patients with AIS were consecutive patients indicated for endovascular treatment with large vessel occlusion. Sampling was done just before intervention similarly to the ACS group. LMWH was administered 1 h before sampling. Blood samples from the control group were obtained from patients undergoing coronary angiography due to non-coronary principal diagnosis; we found nonsignificant atherosclerotic changes on coronary arteries (valve disease, cardiomyopathies, heart failure, atypical chest pain). Again, samples were drawn and processed like samples from patients with ACS, and UFH was administered just before blood sampling. Blood samples were drawn in vacutainer tubes containing EDTA and centrifugated immediately at 4000× *g* for 5 min for 4 °C. The plasma samples were stored in the dark at −80 °C until analysis.

### 4.4. Lipidomic Sample Preparation

A 100 μL aliquot of plasma in an Eppendorf tube was diluted with 300 μL of 2-propanol containing BHT, which resulted in protein precipitation. The tube was vortexed for 10 s, left at room temperature for 10 min, and then centrifuged for 10 min at 14,000 rpm (at 5 °C); 250 μL of the supernatant was then transferred for analysis. Next, 50 μL from every sample was transferred to a 50 mL centrifugation tube to prepare a pooled QC solution. 

### 4.5. Instrumental Conditions

Lipidomic analysis used a U-HPLC (Infinity 1290; Agilent, Santa Clara, CA, USA) coupled to a high-resolution mass spectrometer with a hyphenated quadrupole time-of-flight mass analyzer (6560 Ion Mobility Q-TOF LC/MS; Agilent) with an Agilent Jet Stream (AJS) electrospray (ESI) source.

For lipidomic fingerprinting, an Acquity BEH C18 column (1.7 μm, 2.1 mm × 150 mm; Waters, Beverley, MA, USA) was used for chromatographic separation. The chromatographic system used ESI+ detection and mobile phase A: 10 mM ammonium formate and 0.1% formic acid in acetonitrile:water (60:40, *v*/*v*); mobile phase B was 10 mM ammonium formate and 0.1% formic acid in 2-propanol:acetonitrile (90:10, *v*/*v*). For chromatographic separation of plasma detected in ESI- mode, the following mobile phases were used: A (10 mM ammonium acetate and 0.1% acetic acid in acetonitrile:water (60:40)) and B (10 mM ammonium acetate and 0.1% acetic acid in 2-propanol:acetonitrile (90:10, *v*/*v*)). The flow rate was constant at 0.300 mL∙min^−1^. The column temperature was maintained at 60 °C, and the injection volume was 1 μL. The autosampler was kept at 10 °C. Before injection, the sample injection order was randomized in MS Excel. The QC sample was injected every 10 samples.

The mass analyzer was operated in ESI+ mode under the following conditions: gas temperature 180 °C, drying gas 12 L/min, nebulizer pressure 40 psig, sheath gas temperature 350 °C, sheath gas flow 11 L/min, capillary voltage 3000 V, nozzle voltage 250 V, fragmentor voltage 380 V, and octapole radiofrequency voltage 750 V. Data were acquired over the *m*/*z* range of 50–1700 at the rate of 2 spectra/s. The *m*/*z* range was autocorrected on reference masses of 121.0509 and 922.0098.

The mass analyzer was operated in ESI– mode in the following conditions: gas temperature 180 °C, drying gas 12 L/min, nebulizer pressure 45 psig, sheath gas temperature 350 °C, sheath gas flow 11 L/min, capillary voltage 3500 V, nozzle voltage 250 V, fragmentor voltage 350 V, and octapole radiofrequency voltage 250 V. Data were acquired over the *m*/*z* range of 50–1700 at the rate of 2 spectra/s. The *m*/*z* range was autocorrected on reference masses 119.0363 and 980.0164.

The chromatographic gradient was as follows: For ESI+ mode, the initial composition of 60% A and 40% B was kept from 0 to 2 min, and the initial composition was ramped to 50% A and 50% B from 2 to 4 min, and from 4 to 5 min, it was 60% B. The composition increased to 100% B by 15 min. This was followed by 3 min of initial conditions to re-equilibrate the column. ESI analysis used an initial composition of 60% A and 40% B from 0 to 2 min, and the initial composition increased to 50% A and 50% B from 2 to 4 min, and from 4 to 5 min, it was 60% B. It increased to 80% B by 12 min. The composition was immediately set to 100% B until 15 min to remove triacylglycerols from the column. This was followed by 3 min of initial conditions to re-equilibrate the column.

### 4.6. Processing of Data Generated by Fingerprinting Experiments

The data were processed by the LipidMatch suite [26], which uses MZmine 2 for feature extraction and an R script for lipid identification. A custom-built R script based on the MetaboAnalyst R package was used to filter out features based on their univariate statistics. Statistically insignificant compounds were filtered out if they did not meet the criteria of ANOVA *p*-value < 0.01. These data were then loaded by SIMCA where statistical models were built. Pareto scaling was used to ensure higher significance of low abundant compounds when building PLS-DA models in SIMCA logarithmic transformation. Lipids were identified based on fragmentation spectra and accurate mass in silico libraries, which are part of the LipidMatch suite. Fragmentation spectra of the significant compounds were also compared to those present in METLIN and LIPIDMAPS databases, and their identities were confirmed.

### 4.7. Malondialdehyde Analysis

MDA analysis was based on an already published method [17].

### 4.8. Limitations of the Study

Because of the small number of patients in each group and the cross-sectional nature of the experiment, one must be overly cautious in clinical significance interpretation. Thus, we limited the study as hypothesis generating.

## 5. Conclusions

The findings obtained within this pilot study can be summarized in the following points:

- LC-HRMS/MS lipidomics enabled the detection and identification of approx. 500 lipid species in the studied plasma samples.
- Statistical evaluation of generated data must be carried out carefully, especially with regard to the type of administered heparin, because the extent of neutral lipid hydrolysis largely differs between LMWH and UFH. Lipids such as TG and CE substrates of LPL—together with products of their hydrolysis—had to be eliminated from the dataset to avoid biased results.
- Chemometric assessment of the reduced lipidomic dataset showed that LPIs are significantly different for ACS, diagnosing platelet activation. Higher levels of FAHFAs in controls might be connected to higher anti-inflammatory activity.
- The protective role of FAHFAs as potential biomarkers should be investigated based on larger patient sets.
- Multivariate analysis of lipidomic data is a promising new strategy for discovering the role of lipids in the development and progress of pathologies.

## Figures and Tables

**Table 1:** Significant (VIP) lipids obtained by PLS-DA model on the set of all detected plasma lipids.

Lipid Name	Adduct	Retention Time	*m*/*z*	Fold Change	
				A-K	D-K
FA (20:2)	[M − H]^−^	5.408	307.2636	0.70	0.39
FA (20:3)	[M − H]^−^	4.316	305.2479	0.64	0.31
FA (20:3)	[M − H]^−^	4.669	305.2479	0.73	0.32
FA (20:4).	[M − H]^−^	3.632	303.2324	0.76	0.48
FA (20:5)	[M − H]^−^	2.861	301.2166	0.68	0.39
FA (22:4)	[M − H]^−^	4.741	331.2665	0.74	0.46
FA (22:5)	[M − H]^−^	3.794	329.2481	0.51	0.31
FA (22:5)	[M − H]^−^	4.118	329.2481	0.58	0.31
FA (22:6)	[M − H]^−^	3.235	327.2325	0.58	0.38
FAHFA (14:0/16:2)	[M − H]^−^	3.439	477.3908	0.27	0.08
FAHFA (16:1/18:3)	[M − H]^−^	3.614	529.4225	0.34	0.14
FAHFA (18:1/20:3)	[M − H]^−^	5.205	585.4851	0.52	0.21
FAHFA (18:2/20:4)	[M − H]^−^	3.932	581.4535	0.79	0.41
FAHFA (20:4/18:3)	[M − H]^−^	3.626	579.4376	0.53	0.23
LPC (20:5)	[M + H]^+^	2.239	542.3265	1.23	0.63
LPC (22:5)	[M + H]^+^	2.435	570.3590	1.97	0.48
LPC (22:6)	[M + H]^+^	2.020	568.3428	0.85	0.42
LPC (22:6)	[M + H]^+^	2.450	568.3420	1.24	0.56
LPE (18:2)	[M − H]^−^	2.315	476.2774	1.39	0.67
LPE (20:4)	[M − H]^−^	2.218	500.2774	1.13	0.46
LPE (22:6)	[M − H]^−^	2.092	524.2771	0.97	0.48
LPI (18:1)	[M − H]^−^	2.224	597.3032	2.09	1.13
LPI (18:2)	[M − H]^−^	1.823	595.2878	2.09	1.18
TG (14.0_16.1_20.3)	[M + NH_4_]^+^	12.94	844.7394	0.79	1.78
TG (16:0_16:1_18:0)	[M + Na]^+^	13.33	855.7420	0.80	3.46
TG (16:0_18:2_18:3)	[M + NH_4_]^+^	13.00	870.7544	0.70	1.77
TG (16:0_18:2_22:6)	[M + NH_4_]^+^	12.99	920.7687	0.71	1.49
TG (17:1_17:2_19:0)	[M + NH_4_]^+^	13.85	888.8015	0.73	1.60
FA (16:1)	[M − H]^−^	3.617	253.2168	0.55	0.34
FA (17:1)	[M − H]^−^	4.325	267.2325	0.61	0.35

FA—fatty acid; FAHFA—fatty acyl ester of hydroxy fatty acid; LPC—lysophocholine; LPE—lysophoetanolamine; LPI—lysophoinositol; TG—triacylglycerol.

**Table 2:** Significant (VIP) lipids obtained by the PLS-DA model on the set of polar lipids.

Lipid Name	Adduct	Retention Time	*m*/*z*	PLS-DA VIP	FC A over K	FC D over K	MDA Correlation
FAHFA (14:0/16:2)	[M − H]^−^	3.439	477.3908	2.10	0.27	0.08	−0.437 ***
LPI (20:4)	[M − H]^−^	1.760	619.2877	1.95	1.76	1.06	0.071
FAHFA (16:1/18:3)	[M − H]^−^	3.614	529.4225	1.94	0.34	0.14	−0.411 ***
LPI (18:2)	[M − H]^−^	1.823	595.2878	1.88	2.09	1.18	0.085
LPI (18:1)	[M − H]^−^	2.224	597.3032	1.81	2.09	1.13	0.029
FAHFA (20:4/18:3)	[M − H]^−^	3.626	579.4376	1.72	0.53	0.23	−0.433 ***
FAHFA (18:1/20:3)	[M − H]^−^	5.205	585.4851	1.65	0.52	0.21	−0.359 ***
FAHFA (18:2/20:4)	[M − H]^−^	3.932	581.4535	1.39	0.79	0.41	−0.465 ***
PE (18:1_18:2)	[M − H]^−^	9.196	740.5227	1.27	1.81	1.54	0.157 *
LPE (20:4)	[M − H]^−^	2.218	500.2774	1.24	1.13	0.46	−0.328 ***
PE (16:0_18:2)	[M − H]^−^	9.175	714.5069	1.24	1.90	1.58	0.192 **
LPE (22:6)	[M − H]^−^	2.092	524.2771	1.24	0.97	0.48	−0.340 ***
LPE (18:2)	[M − H]^−^	2.315	476.2774	1.19	1.39	0.67	−0.433 ***
LPC (20:3)	[M + H]^+^	3.948	546.3583	1.17	0.76	0.66	−0.336 ***
PE (16:0_18:1)	[M − H]^−^	9.924	716.5227	1.16	1.72	1.66	0.312 ***
FAHFA (18:0/20:2)	[M − H]−	6.278	589.5155	1.16	1.09	0.75	−0.215 **
LPC (22:6)	[M + H]^+^	2.450	568.3420	1.16	1.24	0.56	−0.348 ***
PC (36:4)	[M + Na]^+^	7.623	804.5551	1.14	0.80	0.98	−0.114
PE (18:0_18:2)	[M − H]^−^	10.08	742.5384	1.14	1.57	1.57	0.192 **
LPC (22:5)	[M + H]^+^	2.435	570.3590	1.13	1.00	0.51	−0.384 ***
LPC (20:5)	[M + H]^+^	2.239	542.3265	1.13	1.23	0.63	−0.330 ***
DMPE (16:0_18:1)	[M − H]−	10.82	744.5537	1.12	1.64	1.55	0.256 ***
PE (18:1_18:1)	[M − H]^−^	9.941	742.5381	1.10	1.67	1.44	0.222 **
LPC (22:6)	[M + H]^+^	2.020	568.3428	1.07	0.85	0.42	−0.382 ***
PC (15:1_20:5)	[M + H]^+^	8.538	764.5249	1.07	1.27	1.64	0.239 ***
PE (34:1)	[M + H]^+^	9.591	718.5409	1.05	1.63	1.89	0.362 ***
LPC (18:3)	[M + H]^+^	1.915	518.3277	1.02	1.30	0.77	−0.230 **

FA—fatty acid; FAHFA—fatty acyl ester of hydroxy fatty acid; LPC—lysophocholine; LPE—lysophoetanolamine; LPI—lysophoinositol; TG—triacylglycerol; DMPE—dimethylphosphoethanolamine; PE phosphoethanolamine; PC—phosphocholine; * *p*-value < 0.05; ** *p*-value < 0.01; *** *p*-value < 0.001.

**Table 3:** List of VIP polar lipids from the PLS-DA model based on female samples only.

Lipid Name	Adduct	Retention Time	*m*/*z*	PLS-DA VIP	FC A-K	FC D-K
FAHFA (14:0/16:2)	[M − H]^−^	3.439	477.3908	1.89	0.18	0.11
FAHFA (16:1/18:3)	[M − H]^−^	3.614	529.4225	1.63	0.29	0.22
LPI (18:2)	[M − H]^−^	1.823	595.2878	1.55	2.50	1.80
FAHFA (20:4/18:3)	[M − H]^−^	3.626	579.4376	1.45	0.46	0.33
LPI (20:4)	[M − H]^−^	1.760	619.2877	1.43	1.98	1.47
FAHFA (18:1/20:3)	[M − H]^−^	5.205	585.4851	1.39	0.45	0.27
Plasmanyl PC (O-16:1/18:1)	[M + CH3COO]^−^	10.14	802.5922	1.33	0.85	1.64
Plasmenyl PC (P-16:1/20:3)	[M + H]^+^	9.725	766.5730	1.32	0.86	1.76
Plasmanyl PC (O-16:0/18:2)	[M + H]^+^	9.729	744.5866	1.31	0.89	1.71
PC (36:4)	[M + Na]^+^	7.623	804.5551	1.29	0.65	1.21
LPI (18:1)	[M − H]^−^	2.224	597.3032	1.20	1.87	1.78
LPC (20:3)	[M + H]^+^	3.948	546.3583	1.14	0.64	0.92
Plasmenyl PS (P-20:0/18:0)	[M − H]^−^	3.922	802.5951	1.10	0.62	0.51
LPC (20:1)	[M + H]	4.024	550.3911	1.08	0.80	1.28
FAHFA (18:2/20:4)	[M − H]^−^	3.932	581.4535	1.07	0.79	0.57
PE (34:1)	[M + H]^+^	9.591	718.5409	1.05	2.02	2.44

**Table 4:** List of VIP polar lipids from the PLS-DA model based on male samples only.

Lipid Name	Adduct	Retention Time	*m*/*z*	PLS-DA VIP	FC A-K	FC D-K
FAHFA (14:0/16:2)	[M − H]^−^	3.439	477.3908	2.81	0.34	0.05
FAHFA (16:1/18:3)	[M − H]^−^	3.614	529.4225	2.58	0.36	0.13
FAHFA (20:4/18:3)	[M − H]^−^	3.626	579.4376	2.28	0.47	0.20
LPC (20:3)	[M + H]^+^	3.948	546.3583	2.25	0.75	0.78
FAHFA (18:1/20:3)	[M − H]^−^	5.205	585.4851	2.02	0.50	0.19
LPE (18:2)	[M − H]^−^	2.315	476.2774	1.87	1.17	0.83
LPC (20:3)	[M + H]^+^	3.630	546.3580	1.78	0.80	0.91
LPC (20:5)	[M + H]^+^	2.239	542.3265	1.75	1.15	0.65
LPC (18:3)	[M + H]^+^	1.915	518.3277	1.74	1.41	0.70
FAHFA (18:2/20:4)	[M − H]^−^	3.932	581.4535	1.73	0.70	0.51
LPC (20:1)	[M + H]	4.024	550.3911	1.64	0.80	0.91
LPE (20:4)	[M − H]^−^	2.218	500.2774	1.59	0.94	0.60
FAHFA (18:0/20:2)	[M − H]−	6.278	589.5155	1.56	0.90	1.01
LPC (18:2)	[M + H]^+^	2.240	520.3443	1.49	1.09	0.69
LPC (22:6)	[M + H]^+^	2.450	568.3420	1.38	1.04	0.62
LPC (22:5)	[M + H]^+^	2.435	570.3590	1.36	0.94	0.55
FAHFA (18:1/18:2)	[M − H]−	4.983	559.4692	1.36	0.86	0.94
FAHFA (18:1/20:3)	[M − H]−	5.205	585.4851	1.31	0.83	0.94
LPC (22:6)	[M + H]+	2.020	568.3428	1.30	0.75	0.49
LPC (22:5)	[M + H]+	2.214	570.3590	1.30	0.87	0.57
FAHFA (16:0/18:2)	[M − H]^−^	4.893	533.4541	1.30	0.82	0.90
LPE (22:6)	[M − H]^−^	2.092	524.2771	1.29	0.95	0.61
LPE (18:1)	[M − H]^−^	2.967	478.2928	1.25	1.22	1.00
LPC (20:2)	[M + H]^+^	3.028	548.3753	1.06	1.02	0.76
LPC (20:3)	[M + H]^+^	2.640	546.3598	1.06	1.02	0.71

**Table 5:** Patient characteristics.

	AIM (*n* = 61) Group A	AIS (*n* = 49) Group D	Control (*n* = 82) Group K	Kruskal–Wallis Test *p*-Value
Age (y)	64	71	64	0.001
Sex (m/f)	41/20	23/26	39/43	−
Clinical characteristics				
Arterial hypertension	35 (57%)	38 (78%)	53 (65%)	−
Diabetes mellitus	13 (21%)	13 (27%)	11 (13%)	−
Current smoker	36 (59%)	10 (20%)	19 (23%)	−
BMI	28.5	28.6	28.1	0.874
Medical history				
History of MI	8 (13%)	5 (10%)	0	−
History of PCI	6 (10%)	2 (4%)	0	−
History of CABG	1 (2%)	3 (6%)	0	−
History of stroke	1 (2%)	0	0	−
Laboratory results				
Creatine (µmol/L)	78	74	73	0.647
Total cholesterol (mmol/L)	4.63	4.4	4.47	0.405
TAG (mmol/L)	1.18	1.39	1.27	0.014 ^a^
LDL cholesterol (mmol/L)	2.88	2.59	2.52	0.008 ^b^
HDL cholesterol (mmol/L)	1.13	1.18	1.37	<0.0012 ^b^
Pre-procedure hypolidimic treatment				
Statin	10 (16%)	14 (29%)	31 (38%)	–
Fibrate	1 (2%)	0	1 (1%)	–
Ezetimibe	0	0	1 (1%)	–
Statin + ezetimibe	0	0	1 (1%)	–
Fibrate + ezetimibe	0	0	1 (1%)	–
Post-procedure hypolidimic treatment				
Statin	60 (98%)	43 (88%)	NA	–
No treatment—patient died	1 (2%)	6 (12%)	NA	–

BMI—body mass index; MI—myocardial infarction; PCI—percutaneous coronary intervention; CABG—coronary artery bypass graft; CKD epi—glomerular filtration; TAG—triacyclglycerols; LDL—low-density lipoprotein; HDL—high-density lipoprotein. Variables are expressed as the median, CI (25%–75%). ^a^ Significant in Dunn’s multiple comparisons test, A vs. D. ^b^ Significant in Dunn’s multiple comparisons test, A vs. K.

## Data Availability

The data presented in this study are available on request from the corresponding author. The data are not publicly available due to ethical reasons.

## Supplementary Materials

The following are available online at https://www.mdpi.com/article/10.3390/metabo11070412/s1, Table S1: ANOVA Post-hoc tests results.
